# Quality of EHR data extractions for studies of preterm birth in a tertiary care center: guidelines for obtaining reliable data

**DOI:** 10.1186/s12887-016-0592-z

**Published:** 2016-04-29

**Authors:** Lindsey A. Knake, Monika Ahuja, Erin L. McDonald, Kelli K. Ryckman, Nancy Weathers, Todd Burstain, John M. Dagle, Jeffrey C. Murray, Prakash Nadkarni

**Affiliations:** Department of Pediatrics, University of Iowa Hospitals and Clinics, 200 Hawkins Drive, 276 MRF, Iowa City, IA 52240 USA; Department of Epidemiology, University of Iowa, Iowa City, IA USA; Institue for Clinical and Translational Science, University of Iowa, Iowa City, IA USA

**Keywords:** Prematurity, Neonatology, Bioinformatics, Data quality, Quality assurance, PEDs data registry, EHR and manual chart abstraction comparison, EHR vs. Manual chart abstraction, and difference in data quality

## Abstract

**Background:**

The use of Electronic Health Records (EHR) has increased significantly in the past 15 years. This study compares electronic vs. manual data abstractions from an EHR for accuracy. While the dataset is limited to preterm birth data, our work is generally applicable. We enumerate challenges to reliable extraction, and state guidelines to maximize reliability.

**Methods:**

An Epic™ EHR data extraction of structured data values from 1,772 neonatal records born between the years 2001–2011 was performed. The data were directly compared to a manually-abstracted database. Specific data values important to studies of perinatology were chosen to compare discrepancies between the two databases.

**Results:**

Discrepancy rates between the EHR extraction and the manual database were calculated for gestational age in weeks (2.6 %), birthweight (9.7 %), first white blood cell count (3.2 %), initial hemoglobin (11.9 %), peak total and direct bilirubin (11.4 % and 4.9 %), and patent ductus arteriosus (PDA) diagnosis (12.8 %). Using the discrepancies, errors were quantified in both datasets using chart review. The EHR extraction errors were significantly fewer than manual abstraction errors for PDA and laboratory values excluding neonates transferred from outside hospitals, but significantly greater for birth weight. Reasons for the observed errors are discussed.

**Conclusions:**

We show that an EHR not modified specifically for research purposes had discrepancy ranges comparable to a manually created database. We offer guidelines to minimize EHR extraction errors in future study designs. As EHRs become more research-friendly, electronic chart extractions should be more efficient and have lower error rates compared to manual abstractions.

**Electronic supplementary material:**

The online version of this article (doi:10.1186/s12887-016-0592-z) contains supplementary material, which is available to authorized users.

## Background

Electronic Health Record (EHR) use can potentially minimize errors, increase efficiency, improve care coordination, and provide a useful source of data for research. Between 2008 and 2013, the proportion of hospitals employing EHRs increased from 9 % to 80 % [1].

For research and quality-improvement purposes, however, data must be extracted from the EHR into an analyzable form. Accurate decisions require correct data, and hence reliable data extraction. Extraction can be done in two ways, manually or electronically. Manual abstraction through visual inspection of patient charts with copy/paste or typing is extremely laborious, and vulnerable to transcription errors, or digit transposition errors due to abstracter fatigue. On the other hand, electronic extraction requires significant Information Technology (IT) expertise, for two reasons:

1. The EHR has a vast number of data elements, which may be recorded as discrete data elements, contained within narrative text, or both. Clinicians must typically collaborate with IT staff to discover the accurate elements and consolidate data from multiple locations in the EHR. To extract the data, IT staff then writes program code in SQL (Structured Query Language [2], the lingua franca of “relational database” technology) which is typically employed for EHR data repositories, and then works with the clinical team to ensure its correctness and completeness.
2. The extracted data typically also require restructuring: for example, the EHR stores all of the thousands of laboratory test results for all patients in a single table, with each row conceptually containing the patient ID, the name of the test, the date/time it was performed, and the value of the result at that point in time. To be analyzable by the typical statistical program, these data must be transformed (again, through programs) into a structure where each laboratory test of interest for a set of patients is placed in a separate column.

While the cost of software-development can be amortized through repeated processing of voluminous data, the primary concern is extraction accuracy.

There have been no studies comparing the accuracy of manual vs. electronic abstraction from EHRs for preterm birth research. The present work performs such a comparison, with the following objectives, which hopefully generalize to other clinical domains:

- To compare electronic vs. manual abstraction for accuracy, in terms of discrepancies or errors, through intensive validation of a subset of variables.
- To understand and categorize the practical challenges in electronic extraction of EHR data, and devise guidelines accordingly for electronic extraction so that datasets from different institutions are comparable.

## Methods

### Data sources

Epic™, the EHR used at the University of Iowa Hospitals and Clinics (UIHC), has been operational since May 2009. Some data (notably laboratory and demographics) were imported from the previous EHR (a system developed in-house) into Epic™ prior to production deployment: laboratory data go back to 1990.

The Prematurity Database at UIHC uses a genetic database application (Progeny™) to store genotypic and phenotypic data collected from maternal interviews and manual chart abstractions from paper and EHR records for 1,772 neonates enrolled after parental consent from 2001 to 2011 (with UIHC Institutional Review Board approval-IRB #199911068 and 200506792). Table 1 summarizes the demographics of the study cohort.

**Table 1:** Neonate demographics

Sex	
Male	55.6 %
Female	44.4 %
Ethnicity	
Non-Hispanic	91.7 %
Hispanic	6.3 %
Unknown/Not reported	2.0 %
Race	
White	85.4 %
African American	6.0 %
Asian	1.9 %
American Indian or Native Alaskan	1.1 %
Other or more than one race	4.2 %
Unknown/Not reported	0.9 %
GA (weeks)	
< 32	44.2 %
32–36	37.7 %
≥37	18.1 %
Mean	32.2
Range	22–42
Birthweights (grams)	
Range	328–5,006
Mean	1,989
Patent ductus arteriosus (PDA)	
% of total Neonates	20.4 %
< 32 weeks	87.6 %
32–36 weeks	12.4 %
≥ 37 weeks	0.0 %

Demographics of the 1,772 neonates enrolled in Iowa’s Prematurity study during the years of 2001–2011

For electronic data abstraction, we investigated variables extracted from Clarity™, the relational data repository from Epic™, whose contents are populated from the production EHR on a nightly basis. Clarity™ allows execution of complex queries returning large sets of data. We extracted data for the same set of neonates, using their Medical Record Numbers (MRNs), along with associated data from 1,444 linked maternal records.

### Analysis

To identify discrepancies, a subset of randomly selected charts was manually reviewed using the production EHR. Using Stata™ version 11, electronically-extracted and Progeny content were compared for accuracy and proper interpretation of data values returned.

### Variables

The variables studied are: gestational age (GA), birth weight (BW), initial white blood cell count (WBC), initial hemoglobin level (Hb), peak total bilirubin level (T Bili), peak direct bilirubin level (D Bili), patent ductus arteriosus diagnosis (PDA), and child race and ethnicity, contrasted with maternal race and ethnicity. The first six variables are numeric, and the last four are categorical, while PDA (a complication of prematurity) is recorded as an ICD-9 code (International Classification of Disease- 9th Revision). For newborns, caregivers enter GA and BW into numeric EHR fields on a birth history page. These data are available only after 2009.

Special considerations for individual variables are described below:

### Gestational age and birth weight

To determine GA for the neonates included in this study, we used an algorithm proposed by Spong [3]. According to this algorithm, for subjects without assisted reproduction technologies (where the conception date is known exactly), 1st and 2nd trimester ultrasound information is used, along with the date of last menstrual period (LMP) if available. For known LMP, if the discrepancy between LMP and ultrasound GA is less than 6 days (for a 1st trimester ultrasound) or less than 11 days (for a 2nd trimester ultrasound) the LMP is used; otherwise the ultrasound GA is used.

As discussed later, the EHR contains much redundant data entered by different caregivers, and not all values entered are identical. To identify all sections in the current version of Epic™ containing information related to GA, we comprehensively reviewed charts of 10 randomly selected neonates with GA <28 weeks, born between 2009 and 2011. We compared data from these sections to electronically-extracted data and the Progeny™ database.

Based on the initial comparison, we studied selected sections for a larger set of 100 neonates with GA <28 weeks. Of these cases, 55 included maternal data and the following sections were reviewed: discharge summary, diagnoses, history and physical, birth history, and the maternal chart and delivery summary.

The discharge summary BW, which is recorded on day of life (DOL) 1, was used as the reference value for identifying errors. The EHR records BW in units of ounces, while Progeny uses grams. We employ a conversion factor of 1 oz = 28.35 gm, considering discrepancies >1 gm to be significant.

### Patent ductus arteriosus

Employing a chart review of 362 of the 512 neonates labeled as having patent ductus arteriosus (PDA), we excluded subjects identified before day of life 3 in which the murmur resolved and/or subsequent imaging showed the ductus arteriosus had closed spontaneously.

### Demographics

Comparing race and ethnicity of the mothers and neonates between the EHR and the manual database identified external database discrepancies. Internal database discrepancies were evaluated by comparing values within the same database, i.e. comparing maternal race to neonatal race.

## Results

Tables 2 and 3 shows the results of analysis for the above variables. It has two sub-tables. Table 2 lists demographic variables, gestational age, and birth weight. Table 3 lists laboratory parameters (First WBC count, initial hemoglobin, peak total and direct bilirubin), and Patent Ductus Arteriosus diagnosis. Details of individual columns are stated below.

**Table 2:** Demographic parameters compared

	1. Manually abstracted database, # of subjects	2. EHR extract-ion, # of subjects	3. Discrepancy (% and # of subjects) between the databases ^a^	4. Manually abstracted database errors	5. EHR-extracted data errors	6. Median discrepancy	7. Discrepancy range
Gestational age	1772	700	2.6 % (18)	1.0 % (7)	1.3 % (9)	1 week	1–10 weeks
Birthweight	1772	735	9.7 % (71)	1.5 % (11)	8.0 % (59) ^c ****^	13 g	2–548 gm
Neonate race ^b^	1758	1384	3.2 % (44)	!-	!-	NA	NA
Neonate ethnicity	1757	596	1.5 % (9)	!-	!-	NA	NA
Mother race ^b^	1749	1378	3.2 % (45)	!-	!-	NA	NA
Mother ethnicity	1739	595	5.0 % (30)	!-	!-	NA	NA

Demographic parameters compared in the paper. The denominator for the percentage is the smaller of the corresponding values in the first two columns

! – EHR manual review data could not be used as a gold standard – often recorded as unknown or null, while the manually collected data was based on patient interviews and was more detailed. *P0.05; **P0.01; ***P0.001; ****P0.0001

^a^ - In general, the sum of the error counts in columns 4 and 5 do not add up to the number in column 3, because the error occurred in both manually and electronically extracted data, or the cause was ambiguous

^b^ - Re-calculated discrepancies after adjusting for the inappropriate Hispanic category in the race column

^c^ - Difference statistically significant, *p* = 4.3 × 10^−9^ by Chi-square test

**Table 3:** Laboratory data and PDA diagnosis compared

	1. Manually abstracted database, # of subjects	2. EHR extract-ion, # of subjects	3. Discrepancy (% and # of subjects) between the databases ^a^	4. Manually abstracted database errors	5. EHR-extracted data errors	6. Median discrepancy	7. Discrepancy range
1^st^ WBC count ^b^	1257	1437	3.2 % (40)	2.5 %(32)	0.6 % (8) ^c***^	0.75 k/mm3	0.01–109 k/mm3
1^st^ Hemoglobin	1333	1460	11.9 % (158)	5.8 % (77)	8.3 % (110) ^d *^	1.4 g/dl	0.1–25.9 g/dl
Peak total bilirubin	1565	1336	11.4 % (152)	6.9 % (92)	5.1 % (68) ^e *^	1.45 mg/dl	0.1–15.2 mg/dl
Peak direct bilirubin	681	674	4.9 % (33)	4.5 % (30)	0.9 % (6) ^f ****^	0.5 mg/dl	0.1–16.4 mg/dl
PDA	512	414	12.8 % (53)	12.8 %(53)	8.2 % (34) ^g ***^	NA	NA

Laboratory data and PDA parameters compared in the paper. The denominator for the percentage is the smaller of the corresponding values in the first two columns

*P0.05; **P0.01; ***P0.001; ****P0.0001

^a^ - In general, the sum of the error counts in columns 4 and 5 do not add up to the number in column 3, because the error occurred in both manually and electronically extracted data, or the cause was ambiguous

^b^ - Re-calculated discrepancies after adjusting for the inappropriate Hispanic category in the race column

^c^ - Difference statistically significant, *p* = 1.3 × 10^−4^ by Chi-square test

^d^ - Difference statistically significant, *p* = 0.012 by Chi-square test

^e^ - Difference statistically significant, *p* = 0.05 by Chi-square test

^f^ - Difference statistically significant, *p* = 4.9 × 10^−5^ by Chi-square test

^g^ - Difference statistically significant, *p* = 0.001 by Chi-square test

- Columns 1–2 show the number of subjects from the manually-abstracted (Progeny™) database and Epic™-extracted data respectively. Note that, in Table 2 the numbers in column 2 are generally smaller than those in column 1, because most data in Epic™ go back only to 2009. In Table 3, on the other hand, some EHR extracted data (WBC, Hb) are more numerous, because the EHR extraction identified data that escaped the manual abstraction process.
- Column 3 shows the number and percentage of patients whose values are discrepant between the manually and electronically-abstracted datasets. The denominator for the percentage is the smaller of the corresponding values in columns 1 and 2.
- Columns 4–5 show the number and percentages of patients with erroneous values in the manually and electronically-abstracted datasets, using chart review as the gold standard. We applied the chi-squared test to determine which differences between errors in manually vs. electronically-extracted parameters were statistically significant. With our data, first WBC count, peak T Bili and D Bili, and PDA had significantly fewer errors with electronic extraction (*p* = 1.32 × 10^−4^, *p* = 0.05, *p* = 4.9 x10^−5^, and *p* = 0.001, respectively). For Race and Ethnicity, these values do not apply, because the manually abstracted parameters were based on detailed patient interviews.
- Columns 6–7 show the median discrepancy and the range of discrepancies between the manual and electronically-extracted values and the chart-review values. Note that several variables (Race, Ethnicity, PDA) are categorical variables, and so “median” and “range” statistics do not apply.

Issues with individual variables are now discussed:

### Gestational age

When comparing the discrepancies of GA, the error rate for the EHR extraction is 1.3 % and the manual abstraction error rate is 1.0 %, indicating that the structured data field extraction is a reliable source, especially considering GA was recorded inconsistently in the EHR at multiple locations. While the median discrepancy was 1 week, two cases were found to be discrepant by 8–10 weeks.

Ultimately eight separate EHR sections were identified where gestational age data were available across the mother and neonates’ chart. The majority of the maternal discrepancies in GA differed from the neonate’s chart by one day. Although 69 % of the time there were discrepancies (range: 1–7 days) in at least one of the fields used to compute GA, only 13 % of the GA values differed by four or more days. A detailed chart of these comparisons can be found in an additional table [See Additional file 1: Table S1].

### Birth weight

The manual and EHR extraction error rates for BW were 1.5 % and 8.0 % respectively. Manual errors were significantly fewer (*p* = 4.3 × 10^−9^). The large number of electronic errors resulted from the extraction algorithm using a manually-entered numeric BW field (implemented post-2009), which is separate from the narrative text of the discharge summary. There appears to be no fixed protocol used by the healthcare provider to enter the numeric BW—different providers appear to copy numbers entered in different parts of the record. However, the median discrepancy difference was a modest 13 grams, which is likely too small to impact population-based research studies.

### Child and maternal race/ethnicity

Many of the race and ethnicity fields in Epic™ were extracted as unknown, null, or patient refused; the fields in the manual database had been populated through patient interviews. Therefore the electronic chart-review data could not be used as a standard for comparison. The original race comparison of the EHR extraction to the manual data database produced large discrepancies (7.8 % in children, 7.0 % in mothers not shown in Table 2).

At the time of our initial analysis some of this difference resulted from definitions. The EHR includes “Hispanic” in the Epic™ race category while “Hispanic” was included only in the ethnicity category in the Progeny database. The categories were adjusted to have similar definitions by moving “Hispanic” into the race category, and the numbers in Table 2 reflect these adjustments.

Intra-database comparisons were also performed to assess if the race of the mother corresponded with the race of the child within both the EHR and Progeny databases (Not shown in Table 2). The EHR and manual discrepancies were 1.5 % and 2.1 %, respectively. Discrepancies were not counted if the data fields contained unknown, null, or patient refused. The neonatal “multiracial” category was rarely selected in the EHR and Progeny databases, 0.6 % and 0.4 % of the time, respectively. Therefore, when the race of the mother did not match the race of the child, it was likely due to not choosing the “multiracial” category for the neonate.

### Laboratory values

Initial comparison of WBC showed a high discrepancy of 108 subjects (results not shown in Table 3). We discovered that most discrepancies came from charts of neonates that were transferred from outside hospitals. The true initial WBC count in these cases is present in scanned and digitized paper-document images in the “media” section of Epic™, which do not undergo optical character recognition (OCR). Consequently, these data are never entered into the EHR’s structured-data fields and cannot be extracted through Clarity™—they can only be accessed by visual (human) inspection of scanned documents.

Therefore, we excluded patients transferred from outside hospitals from the data of 1st WBC in Table 3. After exclusion, there were significantly fewer errors with electronic extraction (*p* = 1.32 × 10^−4^). The remaining electronic errors stemmed from an extraction algorithm issue. The algorithm extracted the first “final result” instead of the first “WBC collected” (which could reflect a preliminary result).

Peak T Bili and D Bili also had fewer errors with electronic extraction (*p* = 0.05 and *p* = 4.9 × 10^−5^). Since these parameters identify the highest value during the neonate’s admission to UIHC, transfer information likely does not affect these data. Initial Hb value, like initial WBC, is affected by transfer data; therefore, the un-adjusted data (in Table 3) favor manual abstraction.

### Patent ductus arteriosus

Neonates were considered to have a physiologic PDA up to DOL 3. A persistent PDA after DOL-3 was considered a complication of prematurity. Manual and electronic abstraction had error rates of 7.7 % vs. 2.6 % (*p* = 0.001). The high discordance is likely because of the absence of a rigorous manual abstraction protocol for recording this parameter. The manually-abstracted database did not record on what DOL the PDA was diagnosed. Of the EHR discrepancies, 72 % of the PDA diagnoses were entered on DOL 0–2, suggesting that a protocol for when to abstract the ICD-9 diagnosis for PDA may improve error rates.

## Discussion

This study is focused on structured data fields specific to preterm birth, but it also highlights obstacles in data extraction that can be translated to all areas of medicine. The present study’s primary limitations are listed below:

1. We explored a modest number of structured data parameters and many of these parameters are specific to studies of preterm birth. Additional structured data such as medications, additional laboratory values, and prenatal maternal diagnoses would be useful to analyze in future.
2. The narrative text fields in the patients’ chart were not included in our data set. As technology advances, extracting data from these areas could be useful in future (see below). Similarly, data captured in the EHR only as images of scanned paper or clinical notes could not be processed: OCR of text is currently insufficiently reliable to be fully automated, and requires painstaking manual proofing.

### EHRs as research data sources: structured vs. textual data

Inducements such as the Electronic Health Record Incentive Program of the Centers for Medicare and Medicaid [4] have made EHRs an important source of data that can be repurposed for research. Wasserman observes that the full potential of the EHR data for pediatric clinical research will only be achieved when research becomes one of the explicit purposes for which pediatricians document patient encounters [5].

EHRs increasingly capture structured data, i.e., discrete elements such as numbers, codes from controlled medical terminologies, and dates. However, data such as symptoms, radiology results, and pathology reports still employ narrative text, which requires Natural Language Processing (NLP) to extract information into a usable structured form. Initiatives such as SHARP [6] aim to provide tools to facilitate NLP as part of an EHR infrastructure. However, today’s state-of-the-art NLP programs are far from 100 % accurate—for example, their accuracy is poor because of the highly abbreviation-filled text of clinical notes [7]. Further, NLP information-extraction programs cannot be reused across all research applications [8]: to achieve high sensitivity and specificity, they must typically be tailored to specific problems, e.g., chronic pain [9], incontinence [10], and asthma [11].

One of the strengths of our study is focusing on using the capabilities of modern EHRs. EHRs allow the design of disease or therapy-specific electronic protocols for data gathering, through templates that support recording of significant positive/negative findings as structured data elements. Instead of using NLP to create our data set for text fields, we utilized the data elements that were already available discretely in the EHR. Using only these data we were able to extract numerous pertinent data parameters. Further protocol use can reduce variation among providers—otherwise, a new intern might omit or fail to elicit certain data when compared to an experienced clinician. Additionally, electronic protocols can provide context and reminders.

While structured fields allow validation in the form of type and range checking, transcription data errors can still occur. Further, EHRs currently lack the sophistication to perform cross-patient validation (e.g., querying the mother’s record while entering data for the baby to ensure that inconsistencies in specific fields do not arise.) Cross-patient linking, if implemented, could also auto-populate numerous fields in the baby’s record.

### Electronic vs. manual extraction of EHR data

Other studies in trauma have corroborated that EHR extraction has given results equal to or superior to manual extraction [12]. The programming effort required to extract structured data from an EHR can be amortized by repeated use of the program with different input parameters—e.g., patient cohorts with different selection criteria. By contrast, the extensive chart reviews described in this report took about 15 min per patient or about 25 h for 100 patient chart abstractions. The human effort and time that can be saved by electronic extraction for large datasets is substantial. For small datasets and rare diseases, manual labor may still be sufficient if it will not outweigh the upfront programming resources needed.

As displayed in Columns 4 and 5 of Tables 2 and 3, in our study data errors were present in both databases that we analyzed. The EHR data extraction error rate, however, was comparable or superior (in the cases of laboratory values and PDA diagnosis) to the traditional manually created database. This is not surprising, since generally laboratory and diagnosis data have the most rigorous input protocols in the EHR. Explanations for the error-rate discrepancies were discussed in the results section of each specific parameter. Below, we discuss general challenges encountered when utilizing electronic data extraction. Guidelines are also derived to address these issues.

- *Definitions of individual data elements may differ between the manual and electronic systems.* This occurred for Race/Ethnicity in this study. The reason for the difference may be historical, or because the two systems may have been developed for different purposes. Sometimes, a system may have transitioned from single selection to multiple selections, but the multiple-selection option may never be used, either because the user interface did not evolve, or because it was too burdensome to recode the existing data. This is the case for Race in the Epic™ EHR: while Epic™ allows more than one race per individual, no actual patient in UIHC’s EHR is labeled with more than one race. Similarly, while Epic™ now has a separate Ethnicity field to record Hispanic status, the older data (which followed US Census usage until the latter was changed) could not be recoded without re-querying the patient.
  - *Guideline*: One must verify that the set of values defining a categorical variable is identical in both datasets, and that the usage is identical. If not, harmonization must be attempted. Sometimes, data conversion using program code can be straightforward, but more often the set of values across the two systems are not fully compatible and will result in data loss upon conversion.

- *The EHR is a longitudinal record.* The same parameter is recorded (with possibly different values) at multiple time points for a given patient. Certain parameter values (e.g., neonatal weight) can change within hours, especially for infants receiving intensive care.

- To complicate matters further, *the same parameter is often recorded redundantly in different sections of the record, often by different caregivers, and EHRs are not organized optimally*. For example, weight for a preterm baby is recorded on delivery, and again on admission to the Neonatal ICU. Thus, when abstracting data one may be comparing values taken from different sections of the record, which were recorded at different times, and are different because the value changed. There are cases where a mother is admitted on one day but delivers her child on a different day, and the gestational age of the child in the note is not updated. (Ideally, the latter would be automatically recomputed.) This impacts intra-EHR consistency; manual processes that require more than one part of the record to be updated may be omitted. Manual transcription errors are not the only cause of a discrepancy between manually-abstracted and electronic data. The EHR design itself results in workflows that necessitate redundant data entry, and invites inconsistencies.
  - *Guideline*: When performing abstraction, usually only one value is picked. The choice of time point varies with the scientific objective—e.g., are we interested in determining incidence of PDA at birth, or PDA that persists past DOL 3? In the latter case, one must also look at other parts of the record, e.g., to check if an echocardiogram was later performed to confirm the diagnosis.

- *All EHR data are not readily computable.* Until an infrastructure (such as envisaged by the Office of the National Coordinator for Health [13]) exists to allow all EHRs to interchange data electronically, data from external laboratories must be manually transcribed or processed with scanning, OCR, or NLP.
  - *Guideline:* Depending on the context and number of parameters in a study, it may be wise to continue to perform manual abstraction: non-reusable, one-time programming efforts may be more expensive than temporary hired labor.

- *The protocol for collection of individual parameters in a manually-compiled dataset may not have been explicitly documented.* Such protocols may not even exist, leading to variation across data-gathering staff. Thus, in our data, PDA was not recorded consistently on DOL 3. The absence of documentation and identical time points for reliable verification makes comparison of EHR vs. manually abstracted data challenging. Similar issues have long been known for parameter measurement in clinical studies, and impact the reliability of future meta-analysis [14]. For example, blood pressure varies with body position, limb used, timing (immediately after showing up at the clinic vs. 10 min later), white-coat effect, etc. Specific protocols can minimize inter-recorder variation.
  - *Guideline*: While one cannot do anything about historically abstracted data, collection protocols must be instituted if not present, and explicitly documented at a per-variable level. Such documentation, or metadata, is an integral component of the dataset, and increases its interpretability. Similar documentation is necessary even for electronic extraction. Otherwise, electronically-extracted datasets from different institutions cannot be compared meaningfully.

## Conclusions

In the management of individual patients, extreme values in observed numerical discrepancies may have less impact than expected because most caregivers are diligent: they mentally over-rule or correct values incompatible with what they observe, or measure the values a second time. Further, the modest median values of the discrepancies indicate that in population-based research, their impact may be minimal.

The EHR used for our study was not specifically enhanced for research applications. Yet the discrepancy range (0.6–8.0 %) observed with electronic extraction of an EHR was comparable to the of manual-abstraction error rate. As EHRs evolve, and healthcare workers become more comfortable using metadata and protocols, extracting data are likely to produce fewer discrepancies than we observed. Historical, non-EHR data, which requires OCR and NLP of scanned notes, remain challenging with respect to output accuracy.

With continued advancements, EHR extractions will largely eliminate transposition and transcription errors that lower the accuracy of manual abstraction.

### Ethics approval and consent to participate

Approval for this project was obtained from the University of Iowa Hospital and Clinic Ethics committee and Institutional Review Board. Specific permission for the use of both Epic and Clarity programs to perform our study was included in the following IRB studies (IRB #199911068 and 200506792). Permission for the access and use of electronic medical records to review private health information was specifically obtained and discussed with our research participants during the consent process.

### Consent for publication

Not Applicable.

### Availability of data and materials

Because of the large amount of individual-level data on numerous variables that was collected for this study, there is a small potential for re-identification of the patients/subjects involved, and therefore we feel that it is not ethically appropriate to make the data publically available for anonymous and unrestricted downloading.

## Additional file

Additional file 1: Table S1. Manually abtracted gestational age across different locations in the EHR. (XLSX 14 kb)

## Abbreviations

BW: birth weight
D bili: direct bilirubin
DOL: day of life
EHR: electronic health records
GA: gestational age
Hb: hemoglobin level
ICD-9: international classification of disease – 9th edition
IT: information technology
LMP: last menstrual period
MRN: medical record number
NLP: natural language processing
OCR: optical character recognition
PDA: patent ductus arteriosus
SQL: structured query language
T bili: total bilirubin
UIHC: University of Iowa Hospitals and Clinics
WBC: white blood cell count
